# Synergic Effect of Borneol and Ligustrazine on the Neuroprotection in Global Cerebral Ischemia/Reperfusion Injury: A Region-Specificity Study

**DOI:** 10.1155/2016/4072809

**Published:** 2016-07-31

**Authors:** Bin Yu, Ming Ruan, Zhen-Nian Zhang, Hai-Bo Cheng, Xiang-Chun Shen

**Affiliations:** ^1^Jiangsu Engineering Laboratory for Research and Industrialization of Empirical Formulae, Nanjing University of Chinese Medicine, Nanjing 210023, China; ^2^Jiangsu Key Laboratory for Pharmacology and Safety Evaluation of Chinese Materia Medica, School of Pharmacy, Nanjing University of Chinese Medicine, Nanjing 210023, China; ^3^School of Food Science, Nanjing Xiaozhuang University, Nanjing 211117, China; ^4^Department of Encephalopathy, Nanjing Hospital of Traditional Chinese Medicine, Nanjing 210001, China; ^5^The Key Laboratory of Optimal Utilization of Natural Medicinal Resources, Guizhou Medical University, Huaxi College Station, Guian New District, Guiyang 550025, China

## Abstract

The cooperation of ligustrazine (LI) and borneol was proved to be much better than each of them in treating cerebral ischemia. However, the mechanism of their synergic therapy is unclear till now. Moreover, whether their cooperation brought different degrees of protection among different brain regions was also unclear. In the present study, the effects of LI, borneol, and their mixture were observed in global cerebral ischemia-reperfusion (GCIR) injury by detecting microcirculation, expressions of caspase-3 and p53, levels of IL-1*β*, IL-6, and TNF-*α*, and contents of SOD, GSH-Px, and MDA in cortex, hippocampus, hypothalamus, and striatum, respectively. Furthermore, Nissl bodies were scored also. Monotherapy of LI or borneol showed obvious improvements in the four regions, specially in cortex and hippocampus. Interestingly, the cooperation of LI and borneol brought some new improvements, specially in hypothalamus and striatum. Thus, the synergic effect of the two drugs showed region-specificity in GCIR injury except the expressions of caspase-3 and p53.

## 1. Introduction

Cerebrovascular disorder comprises a group of neurological diseases, in which ischemic stroke is one of the most severe cerebropathies and considerably impairs patient's life quality. Ischemic stroke accounts for 88% of all strokes and is characterized by rapidly developing clinical signs of disturbance in the cerebral function [1]. Microcirculation disorders are induced by cerebral ischemia, while the onset of reperfusion after cerebral ischemia produces much larger damage because there is a burst of free radical generation in that period [2, 3]. However, ischemia/reperfusion injury (IRI) is more common in cerebrovascular disorders than simple ischemia because of thrombolytic therapies.

Ligustrazine (LI) is one of the most important active ingredients from the traditional Chinese herbal medicine,* Ligusticum chuanxiong Hort*. (Chung Xiong), and is widely used in patients with cerebral ischemia in clinic [4, 5]. However, its brain-protection mechanism is unclear till now.

Nowadays, numerous reports had verified that there was region-specificity in both pathology and pharmacology of central neural system. In different brain regions, the effects of drugs were inconsistent in treating brain disorder [6–8]. Moreover, it has been proved that blood-brain barrier (BBB) does not occur uniformly in all parts of the brain. Some parts around the ventricles are accessible to vital dyes and electron-dense tracers. These areas include the area postrema, median eminence, subcommissural organ, pineal gland, subfornical organ, supraoptic crest, and neurohypophysis [9]. Obviously, it was not suitable any more to research the brain as a whole.

Borneol, a simple bicyclic monoterpene, is from resin of* Dryobalanops aromatica Gaertn. f*. Previous study had demonstrated that the compound displayed obvious neuroprotection, which was involved with the inhibition of proinflammatory factor release [10]. It has been frequently found in many traditional Chinese prescriptions in treating CNS illness, such as Alzheimer disease, stroke, cerebral ischemia, cerebritis, and cerebral edema [11, 12]. Moreover, our previous studies had demonstrated that borneol enhanced the distribution and therapeutic effect of brain-protecting drugs with a region-specificity manner [13–15].

There were preliminary researches which claimed that borneol enhanced the neuron protection of LI in clinic and experimental studies [16–20]. However, it is unclear till now whether the combination still shows cerebral region-specificity protection from ischemia injury and whether the protection is related to the inhibitions of apoptosis, inflammatory reaction, and oxidative injury. In this study, the synergic mechanism of LI and borneol on global cerebral ischemia/reperfusion (GCIR) damage will be researched. Besides, the region-specific characteristic will also be explored.

## 2. Materials and Methods

### 2.1. Materials

LI and BO were purchased from Livzon Pharmaceutical Group Inc. (Shaoguan, China) and Nanjing Pharmaceutical Co., Ltd. (Nanjing, China), respectively. Saline solution and other reagents were obtained from Sinopharm Chemical Reagent Co., Ltd. (Beijing, China).

### 2.2. Animals and Surgical Procedure

**Figure 1:**
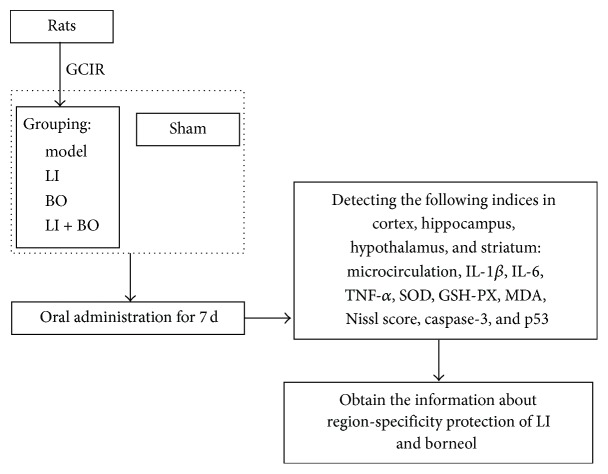
Brief procedure of the study.

Health adult male Sprague Dawley rats (250–300 g) were purchased from Shanghai Slac Laboratory Animal Co., Ltd. (Shanghai, China). The rats were housed in the animal facility of Nanjing University of Chinese Medicine until used. All rats got access to food and water freely under a conditioned environment (12 h light and 12 h darkness cycle, 25°C). The study was performed with the Guide for Care and Use of Laboratory Animals published by the US National Institute of Health (NIH Publication #85–23, revised 1996).  Figure 1 had summarized the study design.

GCIR was induced by 4-vessel occlusion as previous report [21]. Briefly, the rats were anesthetized with 10% chloral hydrate (0.35 g/kg, i.p.); the bilateral vertebral arteries were irreversibly interrupted by electrocauterization through the alar foramina of the first cervical vertebra under anatomical lens. Then the common carotid arteries (CCA) were exposed. After 24 h, the rats were anesthetized by 2.5% halothane and the CCA were clipped by artery clip for 10 min followed by reperfusion. Rats that lost their righting reflex within 60 s and whose bilateral pupils were dilated and lost response to light during ischemia were selected for the experiments. Sham-operated animals were prepared in the same way without electrocauterization of the vertebral artery or occlusion of CCA. All operations were performed under anesthesia condition and their temperatures were maintained at 37 ± 0.5°C using a thermal blanket during surgical procedure.

### 2.3. Grouping and Treating

Forty GCIR rats were randomly assigned to model group, LI group (13.3 mg/kg), BO group (0.16 g/kg), and LI + BO groups (13.3 mg/kg of LI combining with 0.16 g/kg of borneol). Each group comprised ten rats. Both LI and BO were given orally once a day. The sham and model groups were given physiological saline with the volume of 10 mL/kg. After being treated for seven days, they were used for the following detections.

### 2.4. Detection of Microcirculation, Biochemistry, Expressions of Caspase-3 and p53, and Nissl Staining Score in the Four Brain Regions

#### 2.4.1. Microcirculation Measurement

Anesthetized by chloral hydrate solution (0.30 g/kg, ip), the rat was fixed on a stereotaxic frame with a small hole drilled for the implantation of laser Doppler probe, an accessory of MP100 physiological recording system (BIOPAC Systems, Inc., CA, USA). The stereotaxic coordinates of the measuring points were relative to the bregma and dural surface (cortex: AP +3.2 mm, ML +0.8 mm, and DV −3.0 mm; hippocampus: AP −3.8 mm, ML +2.0 mm, and DV −3.0 mm; striatum: AP −2.0 mm, ML +3.0 mm, and DV −3.5 mm; hypothalamus: AP −2.0 mm, ML +0.4 mm, and DV −8.2 mm). After the microcirculations of the four brain regions were detected by the recording system, the rat was sacrificed by decapitation. Then, its brain was taken out immediately, washed by physiological saline, and dried by a filter paper. The cortex, hippocampus, hypothalamus, and striatum were separated by a brush and a surgical knife. Each brain tissue was divided into three parts for the tests of biochemistry, apoptosis, and Nissl staining score below.

#### 2.4.2. Detecting the Contents of IL-1*β*, IL-6, TNF-*α*, SOD, GSH-PX, and MDA in the Four Brain Regions

Brain homogenate (0.1 g/mL) was prepared in ice-cool phosphate buffer by a XHF-D tissue homogenizer (Scientz Biotech Co., Ltd., Ningbo, China) and centrifuged (12000 rpm for 10 min, 4°C). The supernatant was divided into two parts. One of them was used for the measurements of IL-1*β*, IL-6, and TNF-*α* by enzyme linked immunosorbent assay (Elisa). According to the procedure described by Elisa kits manufacturer (Nanjing Jiancheng Bioengineering Institute, Nanjing, China), samples (or standards) and conjugate were added into each well, respectively. After being incubated for 1 h at 37°C, the plate was used to read the optical density value at 450 nm on a Synergy HT microplate reader (BioTek Instruments, Winooski, Vermont, USA). The results were expressed as pg/mL.

The other part was used for the detection of SOD, GSH-PX, and MDA by assay kits (Nanjing Jiancheng Bioengineering Institute, Nanjing, China) according to the manufacturer's protocol.

#### 2.4.3. Detecting the Expressions of Caspase-3 and p53

After brain tissues were homogenized with 1 mM EDTA and 2.5 mL cell lysate, 70 *μ*g of proteins was separated on 12% SDA-PAGE and electrophoretically transferred to PVDF membranes (Pall corporation, NY, USA). The blots were incubated with specific primary antibody against caspase-3 and p53 (Cell Signaling Technology, Beverly, MA, USA) overnight at 4°C. HRP-conjugated second antibody (Santa Cruz Biotechnology, Inc., Dallas, USA) was used for further incubation for 1 h. The blots were washed with TBST. Signals were detected by an enhanced chemiluminescence detection system (Millipore, St. Louis, MO, USA) and analyzed by Image-Pro Plus 7.0 software. Targeted bands were normalized to *β*-actin (KeyGEN Biotech, Nanjing, China) to ensure equal protein loading.

#### 2.4.4. Score of Nissl Staining in the Brain Regions

Brain tissue was removed and postfixed in the fixative for 24 h. After standard dehydration, the tissue was embedded in paraffin, cut into 4 *μ*m sections, and then stained using 1% toluidine blue according to Nissl staining procedure. The image was captured with a microscope (Olympus/IX71, Tokyo, Japan). The number of intact Nissl bodies was counted by full field of five fields randomly.

### 2.5. Statistical Analysis

The data were expressed as mean ± SD. The SPSS 13.0 statistical software was used for standard statistical analysis including one-way ANOVA and Student's *t*-test. A value of *p* < 0.05 was considered statistically significant. Graphical representation was conducted using GraphPad Prism (Version 5).

## 3. Results

### 3.1. Improvement of Microcirculation in the Four Brain Regions of GCIR Rats

**Figure 2:**
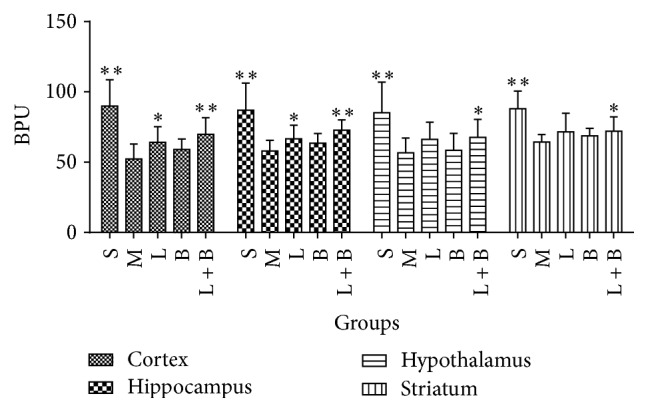
Effects of LI combining with borneol on the microcirculations in the four brain regions of GCIR rats (mean ± SD, *n* = 10). Blood percent unit (BPU) was the unit of blood flow in microcirculation. S, M, L, B, and L + B represented the groups of sham, model, LI (13.3 mg/kg), borneol (0.16 g/kg), and LI + borneol (13.3 mg/kg of LI combining with 0.16 g/kg of borneol), respectively. ^*∗*^ *p* < 0.05 and ^*∗∗*^ *p* < 0.01, compared with the model group.

In model group, microcirculations of all of the four brain regions reduced markedly comparing with sham group (*p* < 0.01), which indicated that microcirculation dysfunction had been formed in model rats. 13.3 mg/kg of LI enhanced the microcirculations in cortex and hippocampus (comparing with model group, *p* < 0.05). 0.16 g/kg of borneol had no obvious improvement on microcirculation in all of the four areas, but it strengthened LI protection in the four regions. Particularly, the combining therapy induced obvious improvement in hypothalamus and striatum (comparing with model group, *p* < 0.05), where neither of the monotherapies showed marked improvement.  Figure 2 displayed the details.

### 3.2. Effects of the Combinations on the Contents of IL-1*β*, IL-6, and TNF-*α* in the Four Brain Regions of GCIR Rats

**Figure 3:**
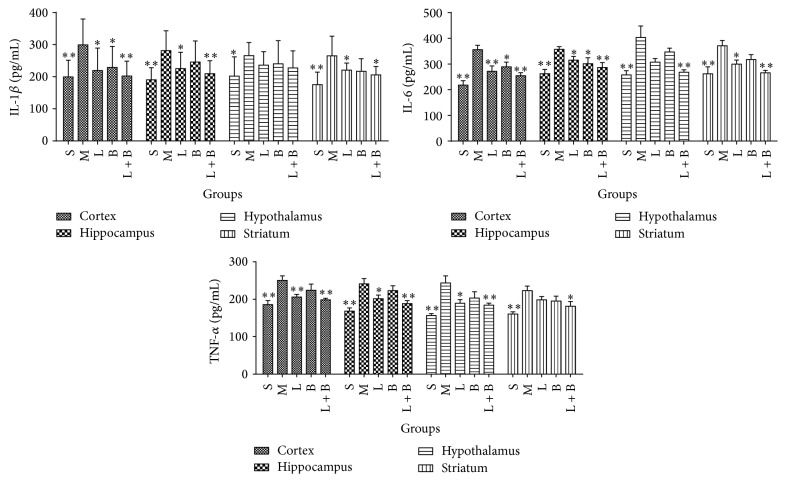
Effects of LI combining with borneol on the contents of IL-1*β*, IL-6, and TNF-*α* in cortex, hippocampus, hypothalamus, and striatum regions of GCIR rats (mean ± SD, *n* = 10). S, M, L, B, and L + B represented the groups of sham, model, LI (13.3 mg/kg), borneol (0.16 g/kg), and LI + borneol (13.3 mg/kg of LI combining with 0.16 g/kg of borneol), respectively. ^*∗*^ *p* < 0.05 and ^*∗∗*^ *p* < 0.01, compared with the model group.

In model rats, the contents of IL-1*β*, IL-6, and TNF-*α* rose markedly comparing with sham group in cortex, hippocampus, hypothalamus, and striatum (*p* < 0.01), which indicated that inflammatory injury had appeared. 13.3 mg/kg of LI decreased levels of IL-1*β*, IL-6, and TNF-*α* in cortex and hippocampus areas, IL-6 in striatum, and TNF-*α* in hypothalamus (comparing with model group, *p* < 0.05, 0.01). Borneol (0.16 g/kg) lessened levels of IL-1*β* in cortex and IL-6 in cortex and hippocampus markedly (comparing with model group, *p* < 0.05). The combining therapy of LI and borneol displayed a much better effect than each of them. Their cooperation inhibited the rise of IL-1*β*, IL-6, and TNF-*α* in all of the four regions (comparing with model group, *p* < 0.05, 0.01), except IL-1*β* in hypothalamus.  Figure 3 displayed the details.

### 3.3. Effects of LI Combining with Borneol on the Contents of SOD, GSH-Px, and MDA in the Four Brain Regions of GCIR Rats

**Figure 4:**
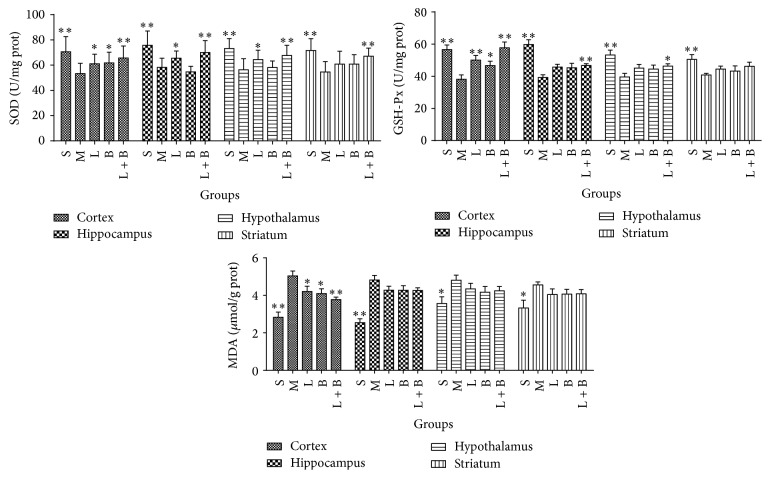
Effects of LI combining with borneol on the contents of SOD, GSH-Px, and MDA in cortex, hippocampus, hypothalamus, and striatum regions of GCIR rats (mean ± SD, *n* = 10). S, M, L, B, and L + B represented the groups of sham, model, LI (13.3 mg/kg), borneol (0.16 g/kg), and LI + borneol (13.3 mg/kg of LI combining with 0.16 g/kg of borneol), respectively. ^*∗*^ *p* < 0.05 and ^*∗∗*^ *p* < 0.01, compared with the model group.

Comparing with sham group, model rats showed marked reduction in SOD and GSH-Px and rise in MDA in the four brain regions of GCIR rats (*p* < 0.05, 0.01), which indicated that oxidative damage had appeared. 13.3 mg/kg of LI improved the disorders of SOD, GSH-Px, and MDA in cortex and SOD in hippocampus markedly (comparing with model group, *p* < 0.05, 0.01). Borneol (0.16 g/kg) increased the contents of SOD and GSH-Px and decreased that of MDA only in cortex region (comparing with model group, *p* < 0.05). However, a better improvement was produced by the cooperation of LI and borneol, which obviously raised SOD in the four areas and GSH-Px in cortex, hippocampus, and hypothalamus and reduced MDA in cortex (comparing with model group, *p* < 0.05, 0.01).  Figure 4 displayed the details.

### 3.4. Effects of LI Combining with Borneol on Scores of Nissl Staining in the Four Brain Regions of GCIR Rats

**Figure 5:**
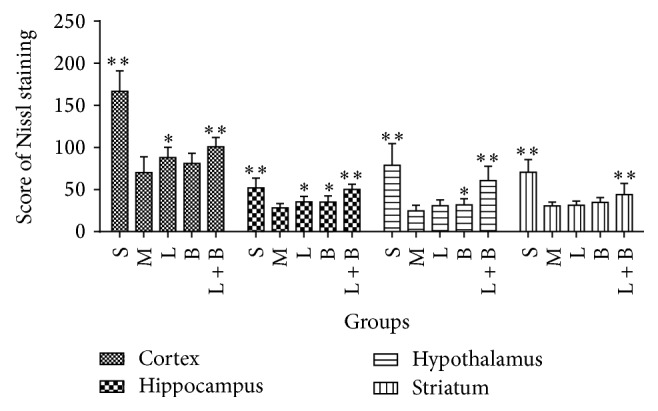
Effects of LI combining with borneol on scores of Nissl staining in the four brain regions of GCIR rats (mean ± SD, *n* = 10). S, M, L, B, and L + B represented the groups of sham, model, LI (13.3 mg/kg), borneol (0.16 g/kg), and LI + borneol (13.3 mg/kg of LI combining with 0.16 g/kg of borneol), respectively. ^*∗*^ *p* < 0.05 and ^*∗∗*^ *p* < 0.01, compared with the model group.

Figure 5 showed the improvement from the combinations on scores of Nissl staining in the four brain regions of GCIR rats. In model group, the numbers of the Nissl bodies in all of the four regions reduced markedly comparing with sham group (*p* < 0.01), which suggested that GCIR process produced wide injury on neurons. 13.3 mg/kg of LI increased the numbers of Nissl bodies in cortex and hippocampus markedly while 0.16 g/kg of borneol protected Nissl bodies in hippocampus and hypothalamus (comparing with model group, *p* < 0.05). In addition, the synergic effect of the two drugs was significant because their cooperation protected the Nissl bodies in all of the four regions (comparing with model group, *p* < 0.01).

### 3.5. Effects of LI Combining with Borneol on the Expressions of Caspase-3 and p53 in the Four Brain Regions of GCIR Rats

**Figure 6:**
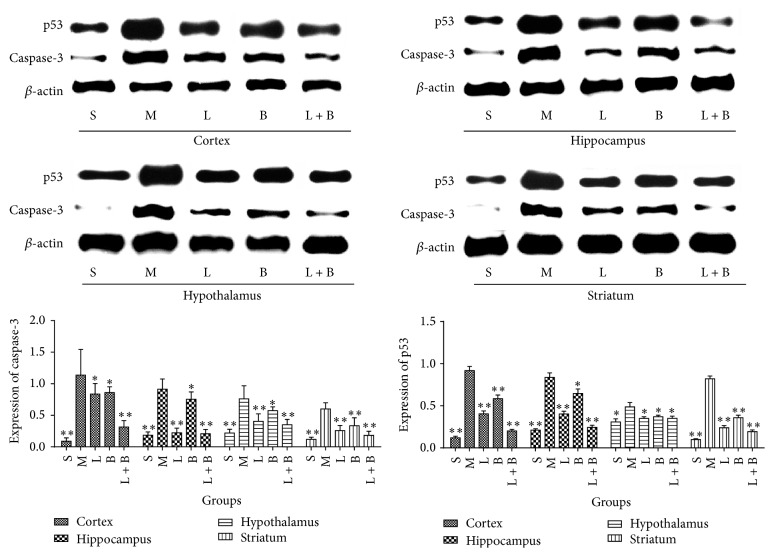
Effects of LI combining with borneol on the expression of caspase-3 and p53 in the four brain regions of GCIR rats (mean ± SD, *n* = 10). S, M, L, B, and L + B represented the groups of sham, model, LI (13.3 mg/kg), borneol (0.16 g/kg), and LI + borneol (13.3 mg/kg of LI combining with 0.16 g/kg of borneol), respectively. ^*∗*^ *p* < 0.05 and ^*∗∗*^ *p* < 0.01, compared with the model group.

As shown in Figure 6, GCIR injury induced the markedly increasing expressions of caspase-3 and p53 in all of the four regions (comparing with sham group, *p* < 0.05, 0.01), which suggested that GCIR process produced apoptosis reaction. Both 13.3 mg/kg of LI and 0.16 g/kg of borneol decreased the expression of two proteins in all of the four areas markedly (comparing with model group, *p* < 0.05, 0.01). In addition, the cooperation of the two drugs had a similar protection to each of them (comparing with model group, *p* < 0.05, 0.01).

## 4. Discussion

Cerebral ischemia-reperfusion attack brings a larger damage than simplex ischemia because the former produces a large number of oxygen-free radicals, which always leads to breakdown of cytomembrane. In addition, early moments of reperfusion also induce other important pathogeneses, such as microcirculation disorder, acute inflammatory cascade reactions, and apoptosis [22–24]. Obviously, cerebral ischemia is a complicated disorder.

At present, the cerebral region-specificity has deeply attracted researchers in exploring mechanisms of pathology and pharmacology. In 2006, it was once reported that iNOS immunoreactivity in cortical penumbra was significantly higher than that in striatum and cortical core following focal cerebral ischemia-reperfusion [25]. The next year, Ho et al. claimed that extracellular signal-regulated kinases (ERK) phosphatase activity was reversibly restrained in cerebral cortex but not affected in hippocampus following ischemic injury, and the desynchrony was probably derived from the different contents of ROS in each brain region [26]. Then, Michalski et al. found region-specific change on the expression of vesicular glutamate in the ischemia-affected brain [7]. Similarly, Noh et al. declared that levels of IL-1b, SRA, TLR2, TLR4, GSH, and mitochondrial complex II/III activities and the number of Iba1-positive microglial cells changed with a region-specific manner in a neurodegenerative disease model [8].

Furthermore, therapeutic effect of central neural system drugs also showed region-specific characteristics. Li et al. found that propofol only regulated the contents of NE in hippocampus and DA in cortex, while the transmitters in striatum areas remained unchanged [27]. In addition, Liao et al. explored the expression of nNOS in different brain areas and proved that the Buyang Huanwu decoction showed more remarkable improvement in caudate putamen and cortex near striatum than that in prepiriform cortex and hippocampus [28]. These researches above indicated that region-specific therapy for cerebropathy has been a potential strategy.

During cerebral ischemia-reperfusion, the activity of free radical scavengers, such as SOD and GSH-Px, remarkably decreases, which leads to brain dysfunction. MDA, one of the most sensitive indicators of lipid peroxidation, increases significantly. So, the levels of MDA, SOD, and GSH-Px are consistent with the severity of cerebral infarction as well as neural recovery [29]. Caspases are intracellular cysteine proteases which mediate cell death and inflammation. Caspase-3 is thought to be tightly linked to the final events in the execution cell death program [30, 31]. The activation of caspase-3 may contribute to the process of apoptosis by changing structures or certain signaling molecules. p53 is proved to be a critical transcriptional activator and exerts lots of biochemical functions in cells. As cellular stress sensor that responds to some signals including ischemia, oxidative stress, and DNA damage, p53 controls process of apoptosis via transcription-dependent and transcription-independent mechanisms to limit the pervasion of injury [32]. Besides the above-mentioned mechanisms, inflammatory response has also an important role in the pathophysiology of brain ischemia, and even it is considered to be a trigger factor for stroke [33, 34]. Cerebral ischemia is thought to be a sterile inflammatory reaction. IL-1*β*, IL-6, and TNF-*α* are the best characterized early response cytokines in the brain and are involved in pathogenesis of ischemic brain injury. In addition, both the microcirculation and score of Nissl staining are important parameters, which directly reflect the cerebral function.

In this study, the neuron protection was evaluated by 13.3 mg/kg of LI, cooperating with 0.16 g/kg of borneol. Monotherapy of LI displayed a better therapeutic effect in cortex and hippocampus than in hypothalamus and striatum areas. In cortex and hippocampus, LI improved microcirculation, enhanced Nissl score and SOD level, and reduced contents of IL-1*β*, IL-6, and TNF-*α*. Moreover, it also increased GSH-Px and decreased MDA in cortex. In hypothalamus, LI increased SOD content and reduced TNF-*α* level. And in striatum, it decreased levels of IL-1*β* and IL-6. Borneol had much better effect in cortex than in the other areas. In cortex, borneol increased the levels of SOD and GSH-Px and reduced contents of MDA and IL-1*β*. In hippocampus, it decreased level of IL-6 and enhanced the Nissl score, while, in hypothalamus, it only enhanced the Nissl score. The cooperation of LI and borneol brought some new improvements, which were not found in monotherapy of the two drugs, in all of the four areas, specially in hypothalamus and striatum. These new improvements included microcirculation, levels of GSH-Px and IL-6 in hypothalamus, microcirculation, levels of GSH-Px, SOD, and TNF-*α* in striatum, and GSH-Px in hippocampus. Obviously, the synergic effect of the two drugs also showed region-specificity protection in GCIR injury. However, the expressions of caspase-3 and p53 did not show markedly region-specificity among the four areas.

It had been confirmed that pathophysiological cascade of cerebral ischemia was involved with a series of stress events which compromised hypoperfusion, inflammation, apoptosis, and peroxide. Besides, systemic thrombolysis had been proved to be an effective therapeutic regimen in ischemia-related neuron damage with the accumulation of the knowledge about the pathophysiological and molecular mechanisms [35]. The present study had revealed a phenomenon of region-specificity protection from LI and borneol, and the mechanism may be related to the different protecting degree on the four regions. The further mechanism will be explored in our future research.

## 5. Conclusion

Our present study had revealed a region-specific protection by treatments of LI (13.3 mg/kg) combining with borneol (0.16 g/kg) in GCIR rats. Although both LI and borneol showed some improvements in the disorder, their cooperation brought much better treatment than each of them. And the mechanisms were involved with improving microcirculation, inhibiting inflammatory response, antioxidative damage, and antiapoptosis process. In addition, the therapeutic effects of CR, borneol, and their combination generally displayed markedly region-specific characteristics.
